# Role of Nutrients Regulating Myeloid Derived Suppressor Cells in Cancer: A Scoping Review

**DOI:** 10.3390/cimb46090549

**Published:** 2024-08-23

**Authors:** Beatriz Pérez-Peláez, Carlos Jiménez-Cortegana, Luis de la Cruz-Merino, Víctor Sánchez-Margalet

**Affiliations:** 1 Department of Medical Biochemistry and Molecular Biology and Immunology, School of Medicine, Clinical Biochemistry Service, Virgen Macarena University Hospital, University of Seville, 41009 Seville, Spain; bpperpel@gmail.com (B.P.-P.); cjcortegana@gmail.com (C.J.-C.); 2 Department of Medicine, School of Medicine, Clinical Oncology Service, Virgen Macarena University Hospital, University of Seville, 41009 Seville, Spain; ldelacruzmerino@gmail.com; 3 Institute of Biomedicine of Seville, Virgen Macarena University Hospital, CSIC, University of Seville, 41013 Seville, Spain

**Keywords:** myeloid-derived suppressor cells, nutrients, vitamins, dietary supplements, neoplasms, cancer

## Abstract

Myeloid-derived suppressor cells (MDSCs) are immature cells with an immunosuppressive function. MDSCs have been related to inflammation in many settings, including infections, transplantation, obesity, aging, or cancer. In oncological settings, MDSCs participate in tumor immunoescape, growth, and metastasis. Certain nutrients can modify chronic inflammation by their interaction with MDSCs. Therefore, the possible influence of certain nutrients on immune surveillance by their actions on MDSCs and how this may affect the prognosis of cancer patients were evaluated in this scoping review. We identified seven papers, six of which were murine model studies and only one was a human clinical trial. Globally, a significant reduction in cancer growth and progression was observed after achieving a reduction in both MDSCs and their immunosuppressive ability with nutrients such as selected vegetables, icaritin, retinoic acid, curdlan, active vitamin D, soy isoflavones, and green tea. In conclusion, the consumption of certain nutrients may have effects on MDSCs, with beneficial results not only in the prevention of tumor development and growth but also in improving patients’ response.

## 1. Introduction

Myeloid-derived suppressor cells (MDSCs) are a heterogeneous population of poorly differentiated myeloid cells with strong immunosuppressive abilities [1]. MDSCs can be granulocytic (G-MDSC) or monocytic (M-MDSC) depending on their lineage [1]. Both subtypes share biochemical features to mediate the suppression of immune responses, such as the upregulation of the signal transducer and activator of transcription 3 (STAT3) or the increase in arginase 1 (ARG1) expression [2]. However, they also use different molecules to carry out immunosuppression. For example, reactive oxygen species (ROS) or peroxynitrite are mainly utilized by G-MDSCs and nitric oxide (NO), while interleukin (IL)-10 or transforming growth factor (TGF)-β are mostly used by M-MDSCs [2]. MDSCs are found in low proportion and regulate the immune system in response to inflammation in normal conditions [3] but are excessively increased and have a strong immunosuppressive activity in many diseases, including cancer, in which MDSCs are involved in multiple stages, including tumor growth and proliferation [4,5].

When chronic inflammation occurs, immature myeloid cells are stimulated by proinflammatory molecules, such as IL-6, IL-1β, IL-17, IL-10, tumor necrosis factor (TNF)-α, or cyclooxygenase (COX)-2, and MDSCs are accumulated in inflammatory sites [6,7] together with other immunosuppressor cells such as regulatory T cells (Treg) or M2 macrophages [8]. However, MDSC development can be re-converted during the early stages of inflammation into dendritic cells and M1 macrophages via interferon (IFN)-γ and TNF-α [9]. Currently, there are a variety of studies focused on the depletion of MDSCs, either promoting their differentiation into non-immunosuppressive mature cells, blocking their immunosuppressive activity, or reducing their concentration in the tumor microenvironment (TME) [10].

Interestingly, it has been suggested that the presence or deficit of nutrients may have effects on inflammation [11] and, consequently, in cancer. The Mediterranean diet, which is characterized by a high intake of fruits, vegetables, whole grains, olive oil, seeds, and moderate consumption of poultry and fish, may be protective in colorectal and breast cancer development [12]. A ketogenic diet is a high-fat, low-carbohydrate diet that induces ketosis and has exhibited antitumor effects in glioma and colon, gastric, and prostate cancers, suggesting its role as a preventive and adjuvant cancer therapy [13]. Low-glycemic index diet consist of consuming low-sugar food such as legumes, non-starchy vegetables or whole grains, and may have favorable effects on colorectal, kidney, and bladder cancers [14]. High-fiber diets are based on fiber-rich foods, such as fruits, vegetables, or whole grains, and play a protective role in the development of breast, endometrial, colorectal, pancreatic, or prostate cancers [15]. Plant-based diets, characterized by the consumption of fruits, vegetables, whole grains, legumes, or seeds, have been associated with improved prognosis in colorectal and breast cancers [16]. Traditional Asian diets include a high consumption of fruits, vegetables, rice, soy, tea, and fish and have been inversely associated with colorectal cancer risk compared to other dietary habits [17]. Anti-inflammatory diets consist of the intake of foods that reduce inflammation, including vegetables, fruits, fatty fish, vegetables, or olive oil. According to the largest meta-analysis of dietary inflammatory index among cancer survivors, the risk of all-cause mortality among patients could be reduced by consuming anti-inflammatory foods after a cancer diagnosis [18]. Lastly, intermittent fasting-based diets, which involve cycling between periods of eating and fasting, should be considered for cancer prevention benefits, but their role in cancer-related metabolic and molecular pathways remains unanswered [19].

Regarding the inclusion of MDSCs in this scenario, high-fiber diets have been demonstrated to promote a larger production of short-chain fatty acids (SCFAs) by the intestinal microbiome, with an anti-inflammatory effect regulated by MDSCs [20]. The ketogenic diet showed not only a reduction in pro-inflammatory cytokines, MDSC, and Treg but also increased the number of CD4+ T cells [21]. In addition, the active form of vitamin D (1,25(OH)2D) plays a role in the regulation of MDSCs by interfering with their differentiation into cells with immunosuppressive activity [22]. Retinoic acid, a vitamin A derivative, also appears to be a regulator of this cellular differentiation in the TME [23]. L-Arginine, which is mainly found in meat, poultry, and fish [24], is vital for T-cell development and function, but it is absorbed by MDSCs to produce Arg1 and immunosuppressive immune responses [25]. Glutamine can be found in fish, poultry, grains, or dairy products [26] and is utilized by MDSCs to exert suppressive effects [27]. Sugars can be either added to meals or naturally presented in foods such as in honey, syrups, and fruit juices [28]. In this sense, a glucose concentration has been found in splenic MDSCs from cancer-bearing mice, which may help to exert their immunosuppressive functions [29]. Also, it has been suggested that antitumor drugs may improve their activity due to the presence of certain nutrients, which may inhibit MDSCs [30].

Altogether, it is of general interest in the field of MDSCs to improve the knowledge and understanding about those nutrient factors that (i) benefit the accumulation of MDSCs in the TME and (ii) limit the acquisition of their immunosuppressive capacity, which is a potential line of investigation towards the development of therapies that prevent tumors from growing. Therefore, we aimed to assess a scoping review to discuss how nutrients could modulate MDSCs in cancer.

## 2. Materials and Methods

### 2.1. Type of Study and Objectives

This is a qualitative review whose general objectives include the following: (i) to describe the effect of different nutrients on MDSCs in cancer and (ii) to answer the PICO question: How do MDSCs modify their activity in the TME when they are under the influence of different nutrients? For this, we searched different studies to (i) identify nutrients that have effects on the proliferation and/or differentiation of MDSCs and (ii) identify dietary patterns that may be associated with better survival rates.

### 2.2. Eligibility Criteria

For this review, we included original studies using the following key terms related to this topic: “Myeloid-Derived Suppressor Cells”, and “Nutrients”, and/or “Dietary Supplements”, and/or “Immunotherapy”, and/or “Diet Therapy”, and/or “Vitamins”, and/or “Neoplasms” and/or “Cancer” and/or “Tumor”. Studies should have been published between 2015 and 2022 and written in either English or Spanish.

Those studies that were different from the original articles (e.g., conference abstracts, editorials, letters, or books, which did not report novel findings), written in a different language from English or Spanish (due to the difficulty in the comprehension of those studies), published before 2015 (since we did not find studies addressing the impact of nutrients on MDSCs before that year), and without full text access (due to the necessity of accessing the complete manuscript to verify the obtained results) were excluded from this scoping review.

## 3. Results

### 3.1. Screening of Original Articles

**Figure 1:**
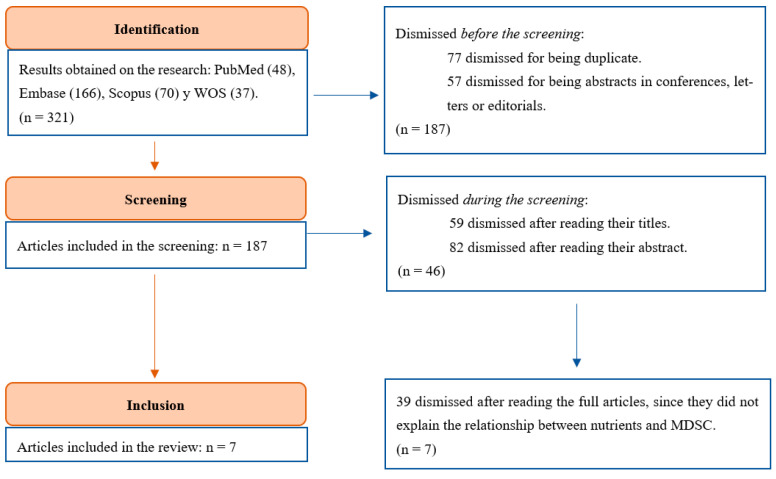
Article selection process according to the PRISMA guidelines.

We applied the PRISMA guidelines for systematic reviews. The process is represented in Figure 1. After the search of the key terms mentioned in Section 2.2, we found a total of 321 results (48 from PubMed, 166 from Embase, 70 from Scopus, and 37 from WOS). We excluded 57 (21 from PubMed, 33 from Embase, 3 from Scopus, and 0 from WOS) of them because the studies were not original articles. Also, we removed 77 duplicates, resulting in a total of 187 articles remaining.

Next, we focused on the relation of the studies to the research question. A total of 59 and 82 studies were excluded because they did not meet this inclusion criteria according to their title and their abstract, respectively. After carefully reading the remaining 46 articles, we realized that 39 of them did not answer the research question properly. Therefore, we finally included seven articles in this review.

### 3.2. Selected Original Articles

After the screening process, we found seven original articles whose main methods and findings are summarized in Table 1.

Hui-Ming Chen et al. [31] determined the effects of a mixture of selected vegetables (SVs) on the antitumor activity of lung cancer-bearing mice. The SVs include soybeans, mushrooms, mung beans, red dates, scallions, garlic, lentils, leeks, Hawthorn fruit, onions, ginsengs, angelica root, licorice, dandelion root, senegal root, ginger, olive, sesame seeds, and parsley. In the study, mouse models had Lewis cells lung cancer (LLC) with NK cell depletion after a subcutaneous injection of ovalbumin-marked tumor cells and anti-CD49b antibodies, as well as lung cancer with liver metastasis after an injection of tumor cells in the liver. Both murine models were treated with a mixture of SVs, starting 10 days before the injection and until the day of sacrifice. A control group feeding with a mix of food and water was used. The results suggested that the SV-based diet of the cancer-bearing mice may have antitumoral effects due to a significant delay in tumor growth as well as increased survival compared to the diet of the control group. NK and T cells had a relevant antitumoral role after the SV-based diet, especially in initial tumor stages. MDSC subtypes decreased in both the spleen and the TME, and there was a significant reduction in ArgI in M-MDSCs.

Huimin Tao et al. [32] used orthotopic and subcutaneous models of liver cancer-bearing mice to evaluate the antitumoral activity of icaritin (ICT), a derivative from *Epimedium genus*. Both murine models were treated with ICT. Another group of mice were used as the control, which was fed with corn oil. Experimental groups were intraperitoneally injected with three doses of anti-Programmed cell death 1 (PD-1) in 3-day intervals for 17 days, and they experienced a significant tumor size reduction and improved survival compared to the control group. In addition, in the group of mice treated with ICT, the percentage of cytotoxic T cells significantly increased, and the total number of MDSC and the Arg1-mediated immunosuppressive activity of G-MDSC were reduced compared to the control group.

Long A et al. [33] studied the GD2 molecule in mouse models of osteosarcoma, Ewing sarcoma, and neuroblastoma. The authors also evaluated the effects of all trans-retinoic acid (ATRA) combined with anti-GD2 chimeric antigen receptor (CAR)-T therapy. GD2-CAR-T was applied between 3 and 5 days later with a complementary anti-IL-17 intraperitoneal therapy 3 days per week. ATRA 5 mg was subcutaneously administered every 21 days. GD2 expression was found in 100% of osteosarcoma-bearing mice and in some cases of Ewing sarcoma and rhabdomyosarcoma murine models. In the sarcoma model, high levels of MDSCs (especially G-MDSCs) were observed compared to the neuroblastoma model, and this cell population was depleted using GD2-CAR-T therapy. Similar results were obtained using ATRA.

Rui K et al. [34] evaluated the effects of curdlan (a glucan used in the food industry as a thickener) on MDSCs from LLC-bearing mice. Intragastric curdlan was administrated every 2 days for one week, and then LLC cells were injected subcutaneously. MDSCs were detected via dectin-1 receptor from curdlan. Curdlan-treated mice significantly improved the differentiation of MDSCs in both the spleen and the TME. Also, the Arg1 and NO levels were notably reduced, which was in line with the significant reduction in tumor growth.

Lesinski G et al. [35] carried out a phase II clinical trial with crossover design to evaluate the efficacy and pharmacokinetic of soy phytochemicals. Thirty-two prostate cancer patients with Eastern Cooperative Oncology Group Performance Status (ECOG) 0–1, asymptomatic tumor biochemical recurrence, and without evidence of disease in complementary tests were enrolled. After a control to limit the consumption of soy phytochemicals, patients received two slices of soy-enriched bread (34 mg of soy isoflavones per 50 g) for 8 weeks. Next, patients consumed a diet of legume-free food for 2 weeks and later received two slices of soy and almond-enriched bread for 8 weeks. NK cells significantly increased, and Th1-associated cytokines, Treg, and MDSC were significantly reduced after the diet compared to basal levels.

Fleet J et al. [22] studied the role of 1,25 (OH)2D, the active metabolite of vitamin D, on MDSCs from prostate cancer-bearing mice. Results showed that the levels of VDR were higher in M-MDSCs than in G-MDSCs, as well as higher in the TME compared to peripheric tissues. The levels of CYP27B1 and CYP24A1, metabolic enzymes of vitamin D, were significantly low in not only the MDSC subsets but also all tissues. Vitamin D administration increased VDR levels in the MDSCs, suggesting that 1,25 (OH)2D-based therapies may reduce the immunosuppressive function of MDSCs. High levels of NOs2 and Arg1 were also found in the TME and correlated with the proportion of MDSC.

Xu P et al. [36] evaluated the activity of epigalocatequin-3-galato (EGCG) from green tea in MDSCs from mice bearing 4T1 breast cancer cells. Mice were divided into six groups depending on the dose of EGCG—250 μg/mL, 500 μg/mL, 1000 μg/mL, and 2000 μg/mL—as well as sterilized water in the remaining two groups. One month after treatment, 4T1 cells were subcutaneously injected in all groups, except the remaining fed with sterilized water and without 4T1 cells injected, which was used as control. EGCG significantly delayed tumor growth and reduced the number of MDSCs compared to the control group. Also, after MDSC isolation, mice were treated with EGCG and experienced a reduced expression of the genes related to MDSC proliferation.

## 4. Discussion

Research focused on novel antitumor therapies is constantly growing. Currently, immunotherapy is one of the most promising research areas in oncology [37]. There are multiple types of therapies to boost the function of the immune system, including those targeting immunosuppressor cells, such as MDSCs [10]. Many studies are focused on elucidating the mechanisms that MDSCs use to promote cell growth and proliferation.

In addition, there are a variety of risk factors that promote cancer, including tobacco or obesity (and unhealthy diets), which also promote the proliferation and accumulation of MDSCs within the TME [38,39]. On the contrary, based on the literature from the section *Results*, nutrients may positively impact MDSC depletion, which has been associated with a diminished immunosuppressive activity and correlated with higher levels of cytotoxic cells, such as NK or CD8+ T lymphocytes. The studies that have been analyzed in this review have evaluated the functionality and levels of MDSCs in not only the TME but also the peripheral tissues, suggesting that nutrients may have a key role in chronic systemic inflammation via MDSCs. Since chronic inflammation has a role in the development of tumors [40], it could be said that certain nutrients may have a protective role against cancer.

Arginine is an amino acid responsible for antitumor immune responses, and its metabolism is dysregulated in cancer [41]. L-Arginine, which is essential for T-cell development and proliferation [42], is taken up by MDSCs to produce Arg1, which inhibits T-cell responses [43]. Glutamine, another amino acid involved in T-cell differentiation and proliferation [42], is utilized by MDSCs to exert suppressive activities [27]. The targeting of glutamine promotes MDSC apoptosis and the downregulation of MDSC-related molecules, such as indoleamine 2,3-dioxygenase, which produces a deficiency of L-tryptophan and, consequently, T-cell anergy [27,44]. Similarly, glucose concentration has been demonstrated to be higher during MDSC differentiation than in the normal maturation process of myeloid cells, especially due to the overexpression of GLUT1 in MDSCs [29]. Lipid and cholesterol metabolism also play a key role in MDSC differentiation. MDSCs boost their immunosuppressive activity by taking up exogenous fatty acids to carry out a metabolic reprogramming from glycolysis to fatty acid oxidation and oxidative phosphorylation [45]. For example, the fatty acid transport protein 2 (FATP2) is able to control the suppressive activity of G-MDSCs by taking up arachidonic acid [46]. Interestingly, the accumulation of cholesterol in MDSCs seems to reduce the nuclear translocation of LXR-β, which suppresses Arg1 expression in MDSCs and affects their differentiation [47].

Vitamins A, D, and E exert a protective role against certain types of cancer due to its positive impact on immune function, antioxidant defense, inflammation, and epigenetic regulation, which ultimately influence cell behavior and counter stress and DNA damage [48]. These vitamins have also been shown to improve MDSC differentiation into mature cells [49]. ATRA treatment has substantially activated the ERK1/2 pathway and specifically upregulated the glutathione synthase gene expression and the accumulation of glutathione in pMDSCs thus depleting this cell population [50]. Vitamin E has been demonstrated to reduce the levels of ROS and NO, as well as inhibit prostaglandin E2 (PGE2) and COX2, which ultimately affect MDSCs [51]. Vitamin D and its analogs have been widely studied. This vitamin has demonstrated anti-proliferative, pro-apoptotic, and pro-autophagic effects [52] that are related to VDR expression, which progressively decreases as tumors progress [53,54]. The anti-proliferative action of vitamin D has been attributed to its effect on cell differentiation and the exit of the cell to G0 [54,55], which is due to the increased expression of p21 and p27 and a lower expression of C-MYC30. The pro-apoptotic effect has been related with a reduced expression of Bcl-2 and Bcl-XL and the increased expression of pro-apoptotic proteins, such as Bax, Bak and Bad [54]. Conversely, calcitriol has reduced systemic inflammation via COX-2 and PGE2 inhibition, as well as the production of pro-inflammatory cytokines [54]. Vitamin D has antioxidant effects due to the expression of ROS-related enzymes, such as superoxide dismutase (SOD)1, SOD2, and the nuclear factor erythroid 2-related factor 2 (NRF2) [54]. We recently found that a vitamin D deficiency in diffuse large B-cell lymphoma patients has been associated with a lack of treatment effect, correlating with high levels of MDSCs and tumor progression [56].

Although most of studies have established the effect of supplementation with individual nutrients, the combination of nutrients could bring potential benefits. However, it would be difficult to determine whether the effects are due to one nutrient or the synergic effects of all of them [31]. Other investigators have suggested that the benefits were caused by the interaction of diet with other elements. Xu P et al. [36] realized that EGCG reduced the immunosuppressive effect of MDSC in vitro and in vivo, but in vivo settings required a lower dose than in vitro assessments due to the presence of other elements that participate in the process.

EGCG is the most abundant catechin in green tea [57] and has shown both antitumor and anti-inflammatory effects [58,59]. Conversely, black tea seems to have less powerful effects [60]. The beneficial effects of EGCC may be attributed to its interaction with specific proteins in different metabolic pathways [60,61]. EGCG has several beneficial properties, including antioxidant effects due to the activity of glutathione peroxidase and the inhibition of oxygen peroxide and NO, which promote a reduction in ROS [62]; anti-inflammatory effects due to the inhibition of pro-inflammatory cytokines; and anti-angiogenic effects due to the inhibition of pro-angiogenic molecules. Mechanistically, EGCG has increased the apoptosis of MDSC by disrupting the Arg-1/iNOS/Nox2/NF-κB/STAT3 signaling pathway, which is essential for immunosuppression thus affecting extracellular matrix–receptor interaction and focal adhesion [36].

The intake of soy isoflavones has been related in Asian countries with a lower risk of developing hormone-dependent tumors and thus a better prognosis [63,64]. Nevertheless, the precise nutrient dose for this protective effect is not still known [65]. Interestingly, isoflavones act on estrogen receptors to decrease their levels, without strong estrogenic effects. Therefore, isoflavones may have beneficial effects on hormone-dependent tumors [65]. Also, the isoflavone genistein has shown anti-angiogenic effects via VEGF downregulation [63].

Curdlan is a D-glucose homopolymer with β(1,3)-glucan used in the food industry that has shown antitumoral activity, among other therapeutic functions [66]. Curdlan interacts with dectin-1 and TLR4 receptors in immune cells from the TME, boosting the antitumor immune response [67]. Curdlan has been demonstrated to promote the differentiation of MDSCs through the NF-κB pathway thus abrogating their suppressive function and improving immune responses [68]. Interestingly, curdlan has also led the differentiation of MDSCs into mature myeloid cells by increasing the expression of CD11c, F4/80, CD40, CD80, CD86, and MHCII, as well as reducing the activity of Arg1 and NO [34].

Importantly, the studies evaluated in this review also analyzed other cells involved in tumor mechanisms, such as Tregs, with immunosuppressive functions, as well as other type of cells with potent cytotoxic antitumor response, such as CD8+ T or NK cells.

Despite the promising benefits of nutrients alone, the real interest may be in their combination with immunotherapies. The PROVIDENCE study suggested a positive impact of vitamin D on outcomes of advanced cancer patients receiving immune-checkpoint inhibitors (ICIs) [69]. Similarly, high doses of vitamin C have showed beneficial effects when combined with ICIs in murine models of mismatch repair–deficient tumors with high mutational burden [70]. SCFAs and polyunsaturated fatty acids have contributed to the maintenance of body weight and fat-free mass in treated cancer patients [71]. Glutamine and arginine supplementation has helped to overcome therapeutic resistance to the checkpoint blockade, which has ultimately improved T cell-mediated immunity [72,73]. Curcumin has also shown interesting results with conventional targeted therapy or ICIs to treat cancer patients [74]. Polyphenols have been promising adjuvant candidates in combination with ICIs via interfering with the PD-1/PD-L1 pathway [75]. Anti–PD-1 therapy, together with a low-fiber diet or probiotics, has shown a lower frequency of interferon-γ–positive cytotoxic T cells in the TME [76]. Conversely, there are some potential conflicts that need further investigation. The use of vitamin E [77], iron intake [78] or selenium supplementation [79] may compromise the effects of immunotherapies to improve antitumor immune responses.

## 5. Conclusions

Despite the low quantity of original studies investigating the effects of different nutrients on MDSCs from mouse cancer models or cancer patients, it has been shown that the inclusion of at least certain nutrients can reduce the number of MDSCs and their immunosuppressive capacity, which may delay tumor growth and progression. However, the real benefits of nutrients in daily clinical practice are still unknown. The next steps should be the development of more in vitro and in vivo mechanistic studies, which will be essential to analyze in more detail the MDSC pathways in which nutrients take part. Similarly, it will be important to develop nutrient-based therapeutic options and establish detailed nutrient profiles in cancer patients, which will be personalized based on each type of cancer and each patient’s characteristics, since clinical responses may vary depending on each patient and the amount of nutrients consumed daily is different. Also, longitudinal studies to evaluate the long-term impact of dietary interventions on MDSCs will be important to monitor response and clinical outcomes, investigate predictive and/or prognostic biomarkers, and even establish possible changes in diets if needed.

It would be very interesting to carry out those studies combining nutrients with cancer immunotherapies, since these combinatory approaches may synergically boost antitumor responses. Those types of studies are not only for the interest of Oncology Departments to guide antitumoral therapies, especially in those cancers with high prevalence such as lung, breast, prostate, and colorectal tumors, but also for the interest of Preventive Medicine Departments to implement certain nutrients in daily diets to reduce the incidence of inflammatory diseases. Specifically, some examples of cancer studies with potential clinical implication may include the following: (i) clinical trials evaluating personalized nutritional interventions to modulate MDSCs, which may include genomic and metabolomic studies to tailor specific dietary approaches that target MDSCs; (ii) the development of nutraceutical supplements for the inhibition of tumor MDSCs; (iii) the design of probiotics that would improve the production of metabolites to deplete MDSCs; (iv) the development of nanoparticle-based delivery systems for specific nutrients that modulate MDSCs, or (v) the evaluation of nutrients as a synergic approach to minimize the immunosuppressive role of MDSCs.

Globally, the main challenge will be to ensure that the human clinical trials are very well-controlled studies, and thus establishing strict inclusion and exclusion criteria to minimize lifestyle-related bias will be essential for this purpose. In addition, patient commitment will be necessary to ensure that results are as reliable as possible.

## Figures and Tables

**Table 1:** Description of selected articles.

Article	Study	Objective	Method	Results
Hui-Ming Chen et al. (2021) [31]	Pre-clinical	Role of selected vegetables on antitumor activity in lung cancer (LC).	LC mouse models with and without liver metastases treated with a mix of selected vegetables.	Significant tumor size reduction and survival improvement in mice fed with selected vegetables compared to control mice. Reduction in G-MDSC percentage and M-MDSC immunosuppressive function.
Tao H et al. (2021) [32]	Pre-clinical	Effects of icaritin on the tumor microenvironment.	Mouse models with orthotopic and subcutaneous liver cancer treated with icaritin and anti-PD-1.	Significant tumor size reduction and survival improvement in mice treated with icaritin compared to control mice. Reduction in G-MDSC levels and activity.
Long A et al. (2016) [33]	Pre-clinical	Effects of GD2-CAR-T therapy and all-trans retinoic acid (ATRA) on solid pediatric tumors.	Mouse models with osteosarcoma, Ewing’s sarcoma, rhabdomyosarcoma, and neuroblastoma treated with GD2-CAR-T and ATRA. The control group received only GD2-CAR-T.	ATRA combined with GD2-CAR-T delayed tumor growth and increased survival. MDSC were also decreased.
Rui K et al. (2016) [34]	Pre-clinical	Effects of curdlan on MDSCs.	Mouse models with Lewis LC treated with curdlan for one week before the subcutaneous injection.	Significant reduction in tumor growth, splenic MDSC, arginase, and nitric oxide.
Lesinski G et al. (2015) [35]	Phase II trial.	Efficacy and pharmacokinetics of soy phytochemicals.	Patients with biochemical relapse of prostate cancer, ECOG 0–1. Diet: (1) two soy-enriched bread slices per day for 8 weeks, (2) Legume-free diet for 2 weeks, and (3) two soy- and almond-enriched bread per day for 8 weeks.	Reduction in Th1, regulatory T cells (Treg), Treg/CD8 ratio, M-MDSCs and MDSC-related cytokines. Increase in natural killer cells.
Fleet J et al. (2020) [22]	Pre-clinical	Role of the active form of vitamin D [1,25 (OH)2D] as a regulator of the activity of MDSCs.	Vitamin D receptor (VDR) KO Mouse models treated with 1,25 (OH)2D.	Vitamin D increased VDR levels and reduced MDSC capacity. Arginase levels were higher in VDR KO mice.
Xu P et al. (2020) [36]	Pre-clinical	Action of EGCG on the activity of MDSCs in breast cancer.	4T1 breast cancer-bearing mice treated with increasing doses of EGCG.	Tumor size was smaller in samples treated with EGCG. This compound improved the expression of cell death-related routes in MDSC and reduced the immunosuppressive capacity of MDSCs.

## Data Availability

Not applicable.
